# Oxygen-Deficient β-MnO_2_@Graphene Oxide Cathode for High-Rate and Long-Life Aqueous Zinc Ion Batteries

**DOI:** 10.1007/s40820-021-00691-7

**Published:** 2021-08-13

**Authors:** Shouxiang Ding, Mingzheng Zhang, Runzhi Qin, Jianjun Fang, Hengyu Ren, Haocong Yi, Lele Liu, Wenguang Zhao, Yang Li, Lu Yao, Shunning Li, Qinghe Zhao, Feng Pan

**Affiliations:** School of Advanced Materials, Peking University Shenzhen Graduate School, Shenzhen, 518055 People’s Republic of China

**Keywords:** Manganese oxides, Oxygen defects, Surface optimization, Aqueous zinc battery

## Abstract

Recent years have witnessed a booming interest in grid-scale electrochemical energy storage, where much attention has been paid to the aqueous zinc ion batteries (AZIBs). Among various cathode materials for AZIBs, manganese oxides have risen to prominence due to their high energy density and low cost. However, sluggish reaction kinetics and poor cycling stability dictate against their practical application. Herein, we demonstrate the combined use of defect engineering and interfacial optimization that can simultaneously promote rate capability and cycling stability of MnO_2_ cathodes. β-MnO_2_ with abundant oxygen vacancies (V_O_) and graphene oxide (GO) wrapping is synthesized, in which V_O_ in the bulk accelerate the charge/discharge kinetics while GO on the surfaces inhibits the Mn dissolution. This electrode shows a sustained reversible capacity of ~ 129.6 mAh g^−1^ even after 2000 cycles at a current rate of 4C, outperforming the state-of-the-art MnO_2_-based cathodes. The superior performance can be rationalized by the direct interaction between surface V_O_ and the GO coating layer, as well as the regulation of structural evolution of β-MnO_2_ during cycling. The combinatorial design scheme in this work offers a practical pathway for obtaining high-rate and long-life cathodes for AZIBs.

**Supplementary Information:**

The online version contains supplementary material available at 10.1007/s40820-021-00691-7.

## Highlights

- The concurrent application of vacancy enrichment and surface coating in β-MnO_2_ electrode can both improve the intercalation kinetics and inhibit the Mn dissolution.
- The oxygen-deficient β-MnO_2_@graphene oxide electrode delivers a reversible capacity of 129.6 mAh g^−1^ after 2000 cycles at 4C, outperforming the state-of-the-art MnO_2_-based cathodes.
- The excellent performance is rooted in the strong binding of graphene oxide on defective β-MnO_2_ and the regulated structural evolution into the Zn_x_Mn_2_O_4_ phase.

**Supplementary Information:**

The online version contains supplementary material available at 10.1007/s40820-021-00691-7.

## Graphical abstract

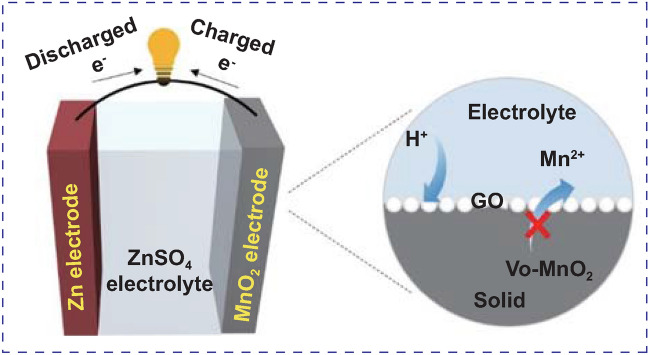

## Introduction

The worldwide transition from fossil fuels to sustainable energy sources has spawned a rising demand for more reliable and low-cost batteries in the field of large-scale energy storage [1], where safety and economic issues are more of a concern than energy density. Rechargeable aqueous zinc ion batteries (AZIBs) [2], because of their non-flammability, cost effectiveness, environmental benignity, and abundant sources, offer a promising alternative to the lithium-ion battery technology in stationary grid-connected applications. Currently, the performance of AZIBs is largely limited by available cathode materials, of which renowned examples include manganese oxides [3], vanadium oxides [4], Prussian blue analogs [5, 6], and organic species [7]. Among them, polymorphs of MnO_2_ have captured particular attention due to their outstanding theoretical capacity and a preferable theoretical voltage versus Zn anode [8–12]. However, the development of MnO_2_ cathodes has been impeded by scientific challenges related to the kinetic limitations and capacity fading, which can be ascribed to the sluggish Zn^2+^ diffusion in the cathode [13] and the irreversible phase transformation [14], respectively. To realize high-rate and long-life AZIBs, it is therefore required to formulate new design strategies for MnO_2_-based cathode materials.

Toward this goal, researchers have adopted various technologies, including pre-intercalation engineering [15], defect engineering [16, 17], interfacial optimization [18, 19], and metal-doping [20], etc. Especially, the incorporation of oxygen vacancies (V_O_) is an effective route to improve the rate performance of MnO_2_ electrodes. Previous studies have suggested that electronic conductivity can be enhanced in the presence of V_O_ [21, 22] and that the under-coordinated Mn ions will potentially afford facile transport pathways for ionic charge carriers [23–26]. It is worth mentioning that a recent study of β-MnO_2_ cathode has revealed the massive proton insertion triggered by the introduction of V_O_ into the bulk lattice [27]. These promising aspects enabled by oxygen deficiency may, however, be tarnished by a higher susceptibility to Mn dissolution, which is likely to incur phase transitions. In this regard, surface coating (SC) can be leveraged to inhibit the Mn ions diffusing into the electrolyte. For pristine MnO_2_ cathodes, the benefits of SC have already been demonstrated in several reports with carbon-based coating materials ranging from graphene [28, 29] to polymers [30, 31]. Yet, the pertinent combination of V_O_ and SC has not been explored in AZIB cathodes up to date, despite its fascinating potential to promote rate capability and cycling stability at the same time. Moreover, how such coatings interact with MnO_2_ is rarely discussed, thus depriving researchers of a rational understanding of the role played by SC.

In this work, we report the combinatorial use of defect engineering and interfacial optimization to boost the electrochemical performance of β-MnO_2_ cathode. Electrode with excessive V_O_ and graphene oxide (GO) wrapping is directly synthesized via a simple hydrothermal reaction. V_O_ plays the vital role in facilitating the transfer of electrons and protons, while GO coating suppresses the dissolution of Mn ions. As a consequence, the oxygen-deficient β-MnO_2_@graphene oxide architecture exhibits high capacity, superior charge/discharge rates, and excellent cycle stability. Our work highlights that the tight binding of GO to the surfaces of β-MnO_2_ via the interaction with V_O_ acts in synergy with the regulated formation of spinel Zn_x_Mn_2_O_4_ to guarantee the structural integrity of the electrode during long-term cycling.

## Experimental Section

### Synthesis of β-MnO_2_@GO Nanorods

The β-MnO_2_@GO nanorod was synthesized via a typical hydrothermal method. 30 mL 0.6 M MnSO_4_, 2 mL 0.5 M H_2_SO_4_, and 4 mL 1 mg mL^−1^ GO dispersed aqueous solutions were mixed and continuously stirred for 30 min. 30 mL 0.1 M KMnO_4_ was then added into the resultant solution dropwise, after which the solution was stirred at room temperature for another 30 min and then loaded into a 100 mL Teflon-lined autoclave and maintained at 120 °C for 12 h. Finally, the obtained products were collected by the filter and were washed with deionized water and absolute ethyl alcohol for three times, respectively, and then dried at 80 ℃ for 12 h. The β-MnO_2_ counterpart was synthesized with the same method without adding GO.

### Materials Characterization

The prepared materials were characterized by X-ray diffraction (XRD, Bruker D8 ADVANCE) with Cu Kα radiation. Scanning electron microscopy (SEM, ZEISS SUPRA55) and transmission electron microscopy (TEM, JEM-3200FS) were employed to investigate the micromorphology and microstructure. The thermogravimetric analysis (TGA) data were recorded in O_2_ atmosphere using a 10 ℃ min^−1^ heating rate from 30 to 700 ℃. X-ray photoelectron spectroscopy (XPS, ESCALAB 250Xi) was used to conduct the element composition and electronic structure analysis, in company with the energy-dispersive spectroscopy (EDS, Oxford X-Max 20) and Fourier transform infrared spectroscopy (FTIR). Electron paramagnetic resonance (EPR, Bruker A300-10/12) was performed to characterize the unpaired electron.

### Electrochemical Tests

Electrochemical performance was tested in CR2032-type coin cells which were assembled in air condition. The working cathodes were fabricated by blending active materials, acetylene black (AB) and polyvinylidene fluoride (PVDF) in a weight ratio of 7:2:1 with N-methyl-2-pyrrolidone (NMP) used as a solvent to form a viscous slurry and coat onto Ti foil. The areal active loading for both the β-MnO_2_ and β-MnO_2_@GO is about ~ 2 mg cm^−2^. The as-prepared electrodes were dried in vacuum oven of about 110 ℃ for 24 h. Zinc foil in 10 mm and glass fiber membrane in 16 mm were used as the anode and separator, respectively. The electrolyte contained 3 M ZnSO_4_ and 0.2 M MnSO_4_ in aqueous solution. The LAND-CT2001A battery-testing instrument was conducted for cycle and rate test with assembled cells. EIS was performed on a Chi 660e electrochemical workstation with frequency range from 100 kHz to 0.1 Hz.

### First Principles Calculations

Density functional theory (DFT) calculations were carried out using projected augmented wave pseudopotentials and the generalized gradient approximation in the form of the Perdew–Burke–Ernzerhof exchange–correlation functional modified for solids (PBEsol), as embedded in Vienna ab initio simulation package (VASP). The van der Waals interactions were treated using Grimme’s correction (DFT-D3). To deal with the localization of d electrons on Mn ions, Hubbard-corrected PBEsol+U(+J) functional was employed. More details are given in the Supporting Information.

## Results and Discussion

### Material Characterization

**Fig. 1:**
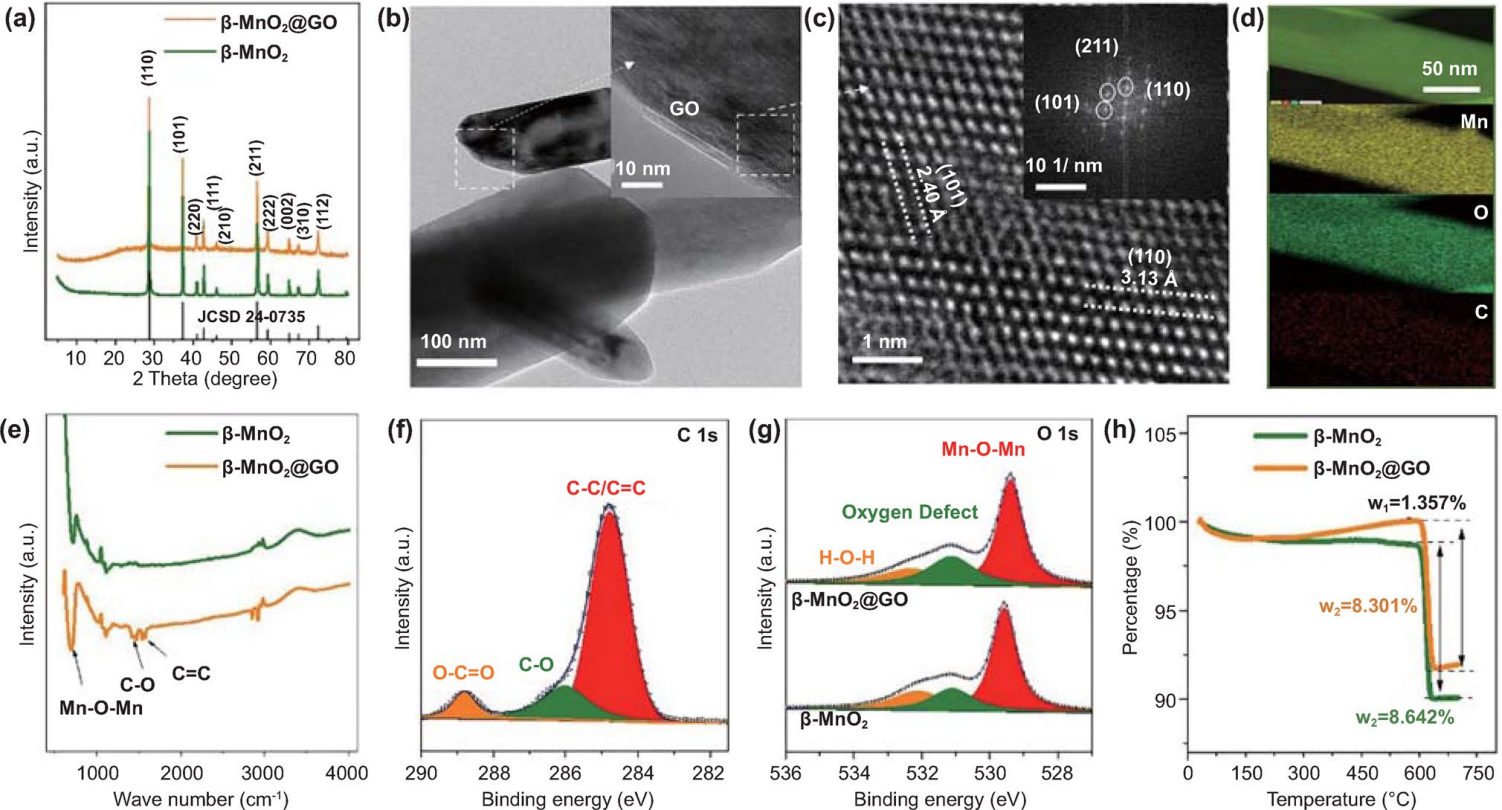
**a** XRD patterns of β-MnO_2_ and β-MnO_2_@GO. **b–d** TEM, HRTEM morphologies, and correlated EDS mapping results of β-MnO_2_@GO (the insets in **b** and **c** show the presence of GO layer and the diffraction pattern, respectively). **e** comparison of FTIR spectra of β-MnO_2_ and β-MnO_2_@GO. **f** XPS peaks of C 1*s* and **g** O 1*s* spectra. **h** TGA curves of β-MnO_2_ and β-MnO_2_@GO in an O_2_-containing atmosphere

XRD patterns of the prepared β-MnO_2_ and β-MnO_2_@GO are shown in Fig. 1a, which match very well with the standard β-MnO_2_ (tetragonal, space group of P42/mnm, PDF #42-0735). This result indicates that GO wrapping does not alter the crystal structure of the β-MnO_2_. SEM results show the nanorod morphologies of β-MnO_2_ and β-MnO_2_@GO with several micrometers in length and 200–300 nm in width (Fig. S1). The high-resolution TEM (HRTEM) confirms the adhesion of GO to the surfaces of β-MnO_2_ (Fig. 1b). Two lattice fringes of (101) and (110) planes are observed for β-MnO_2_@GO (Fig. 1c), with interlayer spacing values of ~ 2.40 and ~ 3.13 Å, respectively, consistent with the XRD results in Fig. 1a. Similar lattice fringe results are also observed for β-MnO_2_ (Fig. S2). There exist some ambiguous areas in β-MnO_2_@GO, which can be ascribed to the formation of a large number of defects.

EDS, FTIR, and XPS further justify the successful wrapping of GO (Fig. 1b) in the β-MnO_2_@GO sample. Figure 1d reveals the uniform distributions of Mn, O, and trace amount of C elements. Comparison of FTIR results in Fig. 1e demonstrates the characteristic peaks of C-O (~ 1432 cm^−1^) and C=C (~ 1576 cm^−1^) [32] in β-MnO_2_@GO. Three clear peaks located at ~ 284.8, ~ 286.0, and ~ 288.8 eV in XPS C 1 s spectrum of β-MnO_2_@GO (Fig. 1f) indicate the existence of C–C/C=C, C–O, and O–C=O bonds, respectively.

The formation of V_O_ can be implied by the XPS O 1*s* spectra (Fig. 1g), where the characteristic peak of V_O_ (~ 531.2 eV) in β-MnO_2_@GO is substantially higher than that in β-MnO_2_. EPR spectra (Fig. S3) showing an apparent symmetrical signal at *g* = 2.0 also suggest the high concentration of V_O_ [33]. The TGA curves of β-MnO_2_ and β-MnO_2_@GO in O_2_-containing atmosphere are shown in Fig. 1h. In the temperature range of 200–600 °C, the TGA curve of β-MnO_2_@GO rises, indicating the filling of V_O_ by oxygen, in contrast to the β-MnO_2_ sample where the mass change is negligible. Here, we cannot rule out the possibility of GO decomposition, which will contribute to mass loss. Due to the formation of more V_O_, Mn ions in β-MnO_2_@GO show lower valence than those in β-MnO_2_, as revealed by Mn 3*s* spectra in Fig. S4. Such a remarkable increase in V_O_ concentration is associated with both the low average oxidation state of Mn (+ 2.7) in the reactant solution, and the deoxygenation of GO during hydrothermal process, which will develop a strong tendency to extract the surface O ions of the as-produced β-MnO_2_ so as to compensate the abundant dangling bonds on the reduced GO. The functional groups on GO may also accelerate the formation of β-MnO_2_, in which case the fast kinetics will potentially give rise to offset from the equilibrium state, for example, in the form of bulk V_O_. This scenario is similar to the cases of TiO_2_@GO [34] and other MnO_2_@GO electrodes in previous report, where the generation of V_O_ in the transition metal oxides can be triggered during their hydrothermal growth in the presence of GO [35–37].

### Electrochemical Performance

**Fig. 2:**
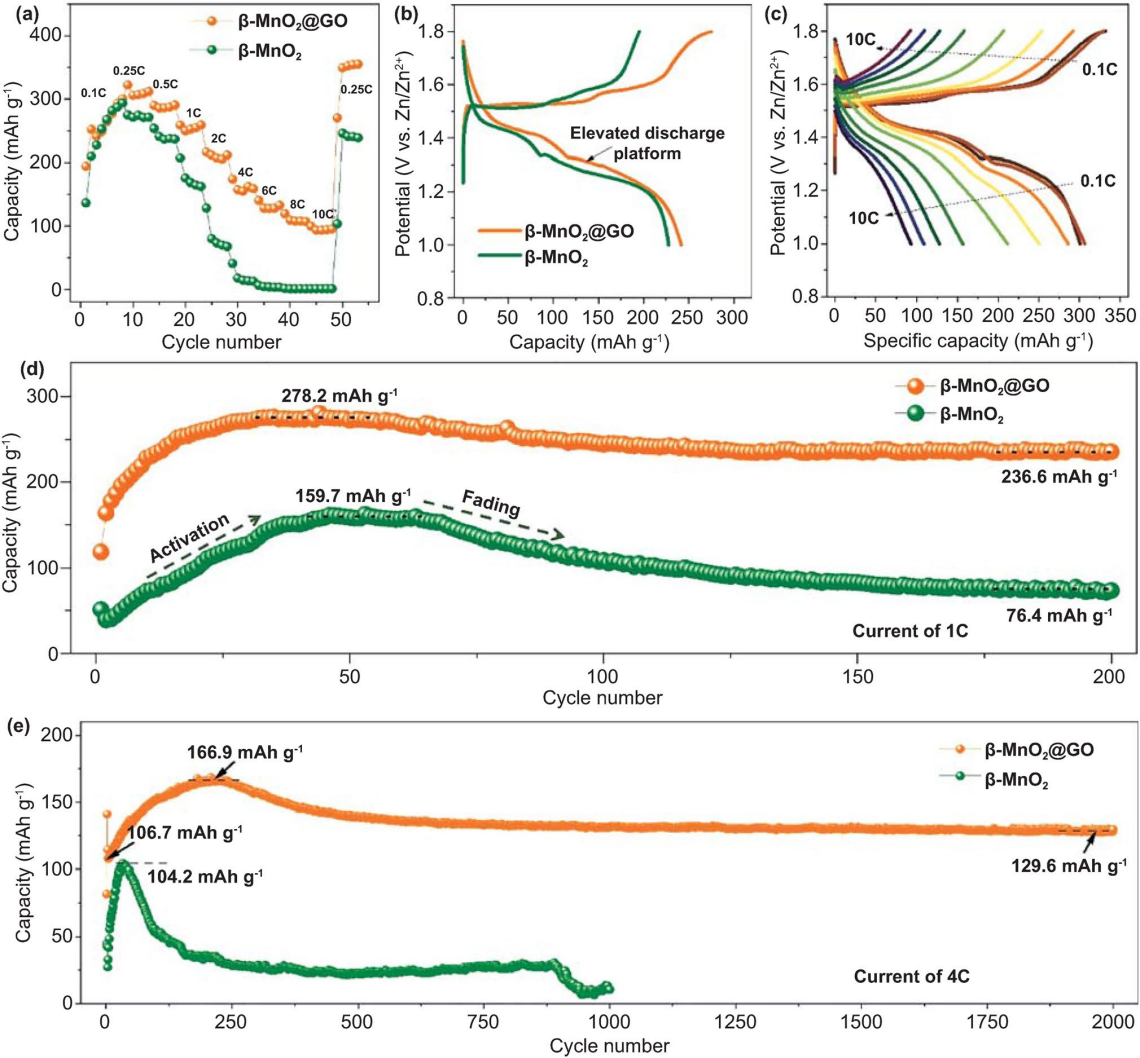
**a**, **b** Comparison of rate performances and the galvanostatic charge/discharge (GCD) curves at the second cycle (at current of 0.1C) of β-MnO_2_ and β-MnO_2_@GO electrodes. **c** GCD curves of β-MnO_2_@GO electrodes at various rate currents. Cycling performances of β-MnO_2_ and β-MnO_2_@GO electrodes at the rate currents of **e** 1C and **f** 4C

Coin-type cells are assembled with Zn plate as anode and aqueous 3 M ZnSO_4_ + 0.2 M MnSO_4_ as electrolyte. The role of the pre-added Mn^2+^ in the electrolyte is to suppress the Mn^2+^ dissolution upon discharge processes, and the optimized Mn^2+^ concentration in electrolyte is ~ 0.2 M (Fig. S5). Figure 2a compares the rate performance of the β-MnO_2_ and β-MnO_2_@GO electrodes. It can be seen that both electrodes show similar capacity activation process in the initial eight cycles at a current of 0.1C (1C = 308 mA g^−1^), indicating that GO wrapping shows little influence on the capacity delivery of β-MnO_2_ at low current rates. After eight cycles, the discharge capacity of β-MnO_2_@GO is stabilized at ~ 322.6 mAh g^−1^. Figure 2b shows the galvanostatic charge/discharge (GCD) curves of the β-MnO_2_ and β-MnO_2_@GO electrodes at a current of 0.1C in the second cycle, indicating that GO wrapping can induce an elevated discharge platform, i.e., a smaller polarization. Figures 2c and S6 show the GCD curves of the β-MnO_2_@GO and β-MnO_2_ electrodes at various current rates, respectively. The discharge capacities of β-MnO_2_@GO are ~ 312.4, ~ 290.9, ~ 259.6, ~ 211.7, ~ 158.6, ~ 132.5, ~ 106.8, and ~ 94.9 mAh g^−1^ at current rates of 0.25, 0.5, 1, 2, 4, 6, 8, and 10C, respectively, which are much higher than those of β-MnO_2_.

Figure 2d, e provides the cycling performances of β-MnO_2_ and β-MnO_2_@GO electrodes at current rates of 1C and 4C, respectively. It can be seen that the cycling performances follow the similar trend: The discharge capacity is activated in the initial cycles and then continuously reduces in the subsequent cycles. This kind of capacity variation is similar to other manganese oxide electrodes in previous reports [38, 39], and the initial capacity activation process can be attributed to the bulk-nanocrystalline evolution during cycling. For β-MnO_2_@GO, at a current rate of 1C, the discharge capacity first increases to ~ 278.6 mAh g^−1^ in 50 cycles and then reduces slowly to ~ 236.6 mAh g^−1^ in 200 cycles. Furthermore, at a current rate of 4C, the discharge capacity first increases to ~ 166.9 mAh g^−1^ in 220 cycles and then reduces slowly to ~ 129.6 mAh g^−1^ in 2000 cycles, with nearly no capacity fading as compared with the initial discharge capacity (~ 106.7 mAh g^−1^). The capacity, rate, and cycling performances of β-MnO_2_@GO are among the best reported manganese oxides (Table S1). Therefore, the combination of V_O_ and GO wrapping on β-MnO_2_ not only enhances the charge/discharge kinetics for superior rate performances, but also improves the cycling stability of the electrode.

### Charge Storage Mechanism

**Fig. 3:**
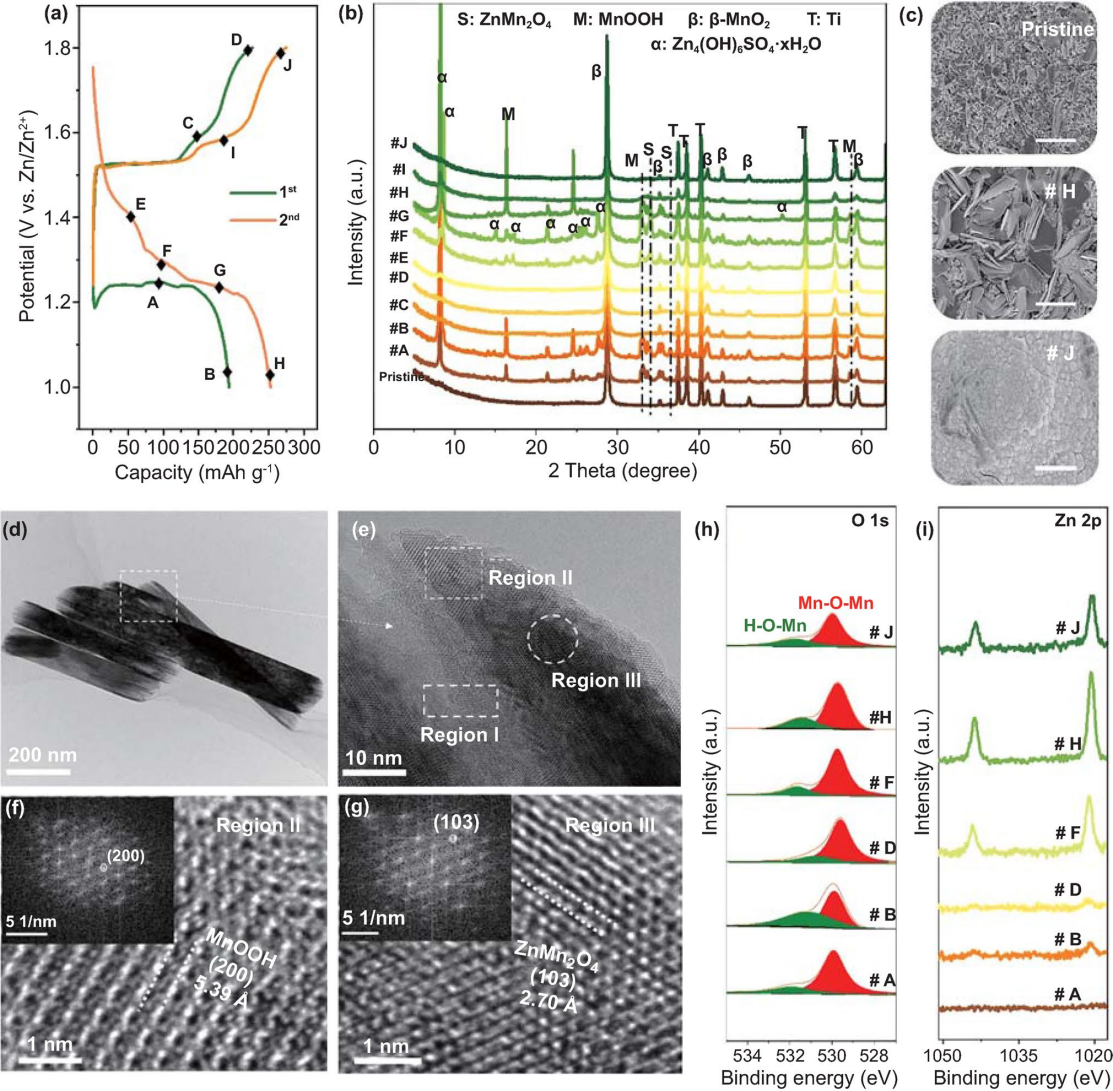
**a** Galvanostatic charge/discharge curves at 0.1C in the initial two cycles, and **b, c** XRD patterns and SEM morphologies of β-MnO_2_@GO electrode at pristine, points #H and #J. **d–g** TEM and HRTEM images of β-MnO_2_@GO at fully discharged state in the first cycle. **h, i** XPS analyses of O 1*s* and Zn 2*p* spectra at selected points. Scale bars in **c** are ~ 2 μm

Insights into the charge storage mechanism is highly significant to understand the enhanced electrochemical performances of β-MnO_2_@GO. Herein, XRD, SEM, TEM, and XPS are comprehensively applied to reveal the charge storage mechanism and the correlated structural evolution of β-MnO_2_@GO upon cycles. Figure 3a shows the GCD curves of β-MnO_2_@GO electrode in the initial two cycles (at current rate of 0.1C), with the correlated XRD patterns at selected states (from point #A to #J) given in Fig. 3b. It can be seen that the (110), (101), (211) peaks of β-MnO_2_@GO located at 28.62°, 37.28°, 56.60° shift negligibly upon discharge/charge processes. After discharge (i.e., at point #B in the first cycle, and point #H in the second cycle), two diffraction peaks at 16.35° and 33.95° emerge, corresponding well to the monoclinic MnOOH (orthorhombic, Pnma (62), PDF #88-0648), a typical product of proton conversion in MnO_2_ [40, 41]. Meanwhile, zinc sulfate hydroxide hydrate by-product (Zn_4_(OH)_6_·ZnSO_4_·xH_2_O, abbreviated as “ZSH,” PDF #44-0673) is generated upon discharge, featuring a set of strong diffraction peaks located at 8.12°, 15.08°, 21.56°, and 24.57°, which is a critical evidence for proton intercalation into the lattice framework of MnO_2_ [20]. The presence of ZSH on the electrode can be further confirmed by the morphology evolutions of β-MnO_2_@GO electrodes, (Figs. 3c, S7), and the detailed analysis for the morphology evolutions is shown in Supporting Information.

As reported previously, for β-MnO_2_, protons rather than Zn^2+^ ions tend to intercalate into the [1 × 1] tunnel framework, owing to the large ion radius and high charge density of the divalent Zn^2+^ ions [24]. Hence, the charge storage in β-MnO_2_@GO is likely to be dominated by the proton intercalation/conversion reactions. When the amount of intercalated proton in surface area exceeds a threshold, it converts to the orthorhombic MnOOH, which explains the co-existence of diffraction patterns of MnOOH (surface area) and protonated β-H_x_MnO_2_ (internal area) phases [27] upon discharge in Fig. 3b. Furthermore, some weak peaks locating at 32.97° and 58.70° are observed upon discharge in the second cycle, which is indexed to the ZnMn_2_O_4_ phase (hetaerolite, 141/amd, PDF No. 24-1133) [3, 42]. HRTEM is also applied to reveal the lattice structures of β-H_x_MnO_2_, MnOOH, and ZnMn_2_O_4_ phases in the discharged electrode, as shown in Fig. 3d–g. The nanorod morphology of β-MnO_2_@GO maintains well upon discharge. We note that the internal part (region I) remains the pristine crystal lattice of β-MnO_2_ (Fig. 3e), while the surface parts (i.e., in region II and III) show a different scenario. The magnified HRTEM images and the correlated diffraction patterns in region II and III (Fig. 3f, g) show the lattice fringes corresponding to (200) plane of MnOOH and (103) plane of ZnMn_2_O_4_, respectively. The detailed analyses of diffraction patterns from regions I to III are illustrated in Fig. S8.

The proton storage behavior in β-MnO_2_@GO can be further confirmed by XPS analyses. Before the XPS tests, the ZSH on electrode is removed by dilute acid to eliminate the influence of by-products. Figure 3h shows the evolution of O 1*s* spectra in initial two cycles, where the peaks near 531.9 eV (referring to Mn–O–H bonds on [MnO_6_] octahedron units) increase upon discharge and decrease upon charge, which is correlated with the regular variation in Mn valence (Fig. S9). Accompanying with the proton insertion/extraction processes, the inevitable Mn^3+^ disproportionation occurs upon discharge. As a consequence, some Mn^2+^ ions dissolve and migrate into the electrolyte, resulting in Mn vacancies on the surface of β-MnO_2_. In the subsequent discharge process, Zn^2+^ ions can easily insert into the defective structure and give rise to the transformation into Zn_*x*_Mn_2_O_4_ (*x* < 1) spinel phase in the surface region.

Figure 3i shows the evolution of Zn 2*p* spectra in the initial two cycles. It can be seen that the Zn 2*p* peaks become obvious starting from the second discharge process (since point #F), indicating that Zn^2+^ cannot insert into the lattice framework of β-MnO_2_ until there are some Mn vacancies generated after the first discharge process. The Zn^2+^ ions in Zn_x_Mn_2_O_4_ are largely unextractable, demonstrating a low reversibility of Zn^2+^ insertion/extraction. Similar charge storage behavior in β-MnO_2_ is also characterized in Figs. S10–S12. Moreover, after long-term cycles, the proton storage reactions still dominate the charge storage of β-MnO_2_@GO electrode (Figs. S13–S15, Tables S2, S3) despite such structural evolution. V_O_ and SC will significantly influence the proton storage behavior and Zn_*x*_Mn_2_O_4_ formation process, which will be discussed in the following part.

### Superior Reaction Kinetics

**Fig. 4:**
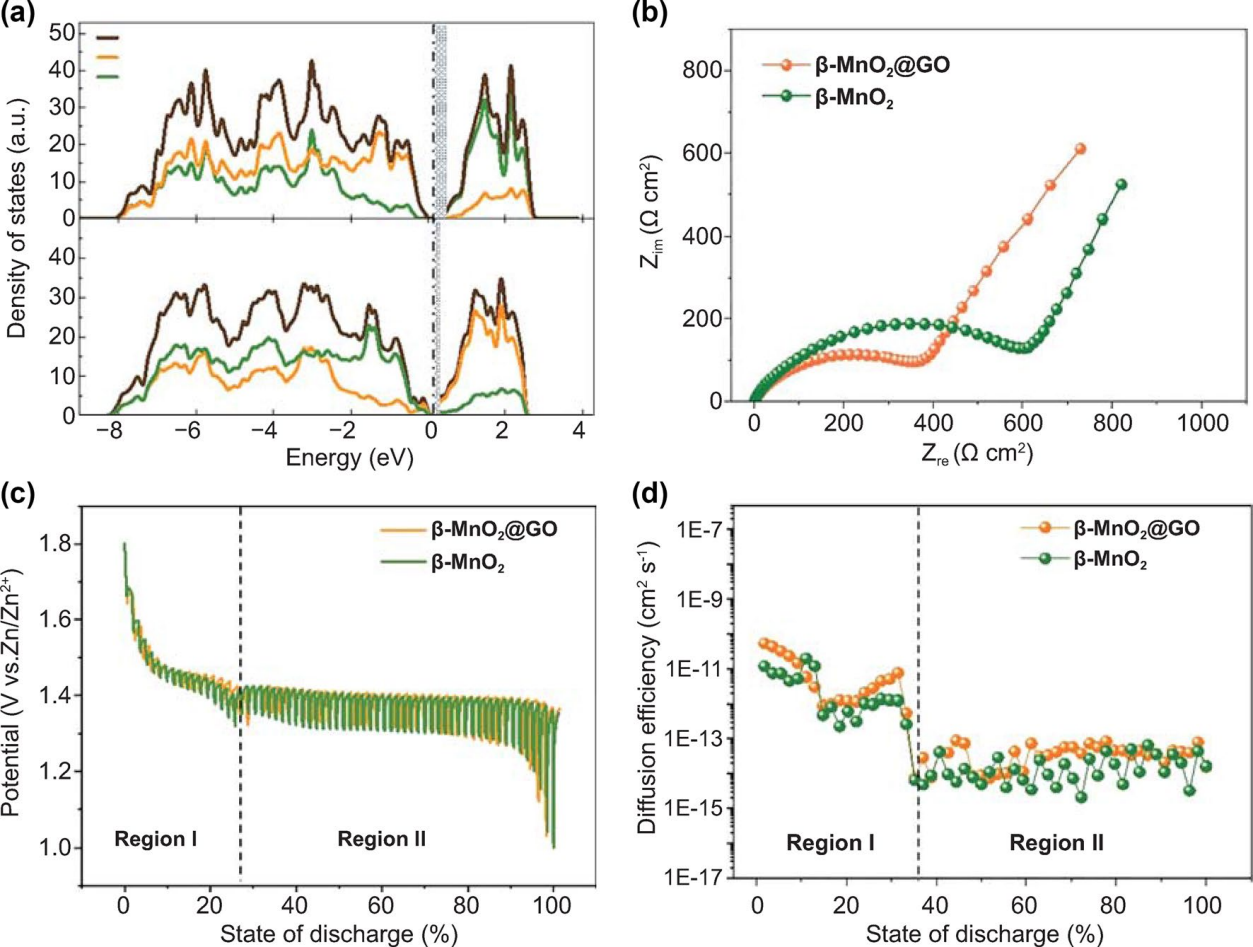
**a** Calculated electron density of states of β-MnO_2_ with and without V_O_. Energies are referenced to the Fermi level. **b** EIS spectra of electrodes with β-MnO_2_ and β-MnO_2_@GO as active materials. **c**, **d** GITT curves and calculated diffusion coefficients

As displayed in Fig. 2a, the boosted rate performance is mainly attributed to the incorporation of V_O_ in β-MnO_2_@GO. Figure 4a shows the calculated electron density of states of β-MnO_2_ and β-MnO_2_ + V_O_ by DFT calculations. The pristine β-MnO_2_ has a bandgap of ~ 0.25 eV, while a lower value of ~ 0.12 eV is obtained after introducing a V_O_ in the supercell, indicating an enhanced electronic conductivity of β-MnO_2_ + V_O_. Consistent with the above result, the β-MnO_2_@GO electrode presents much lower charge transfer impedance of ~ 365.3 Ω cm^2^ when compared with that of the β-MnO_2_ electrode (~ 604.3 Ω cm^2^). The galvanostatic intermittent titration technique (GITT) measurements are further applied to illustrate the proton insertion kinetics (Fig. 4c, d), and the detailed calculation processes of diffusion coefficients are illustrated in SI. The β-MnO_2_@GO electrode shows average diffusion coefficients of ~ 1.13 × 10^−11^ cm^2^ s^−1^ in region I (from 1.8 to 1.35 V) and ~ 4.00 × 10^−14^ cm^2^ s^−1^ in region II (from 1.35 to 1.05 V), which are consistently higher than that of β-MnO_2_ electrode (~ 4.25 × 10^−12^ cm^2^ s^−1^ in region I and ~ 2.57 × 10^−14^ cm^2^ s^−1^ in region II).

### Enhanced Cycling Stability

**Fig. 5:**
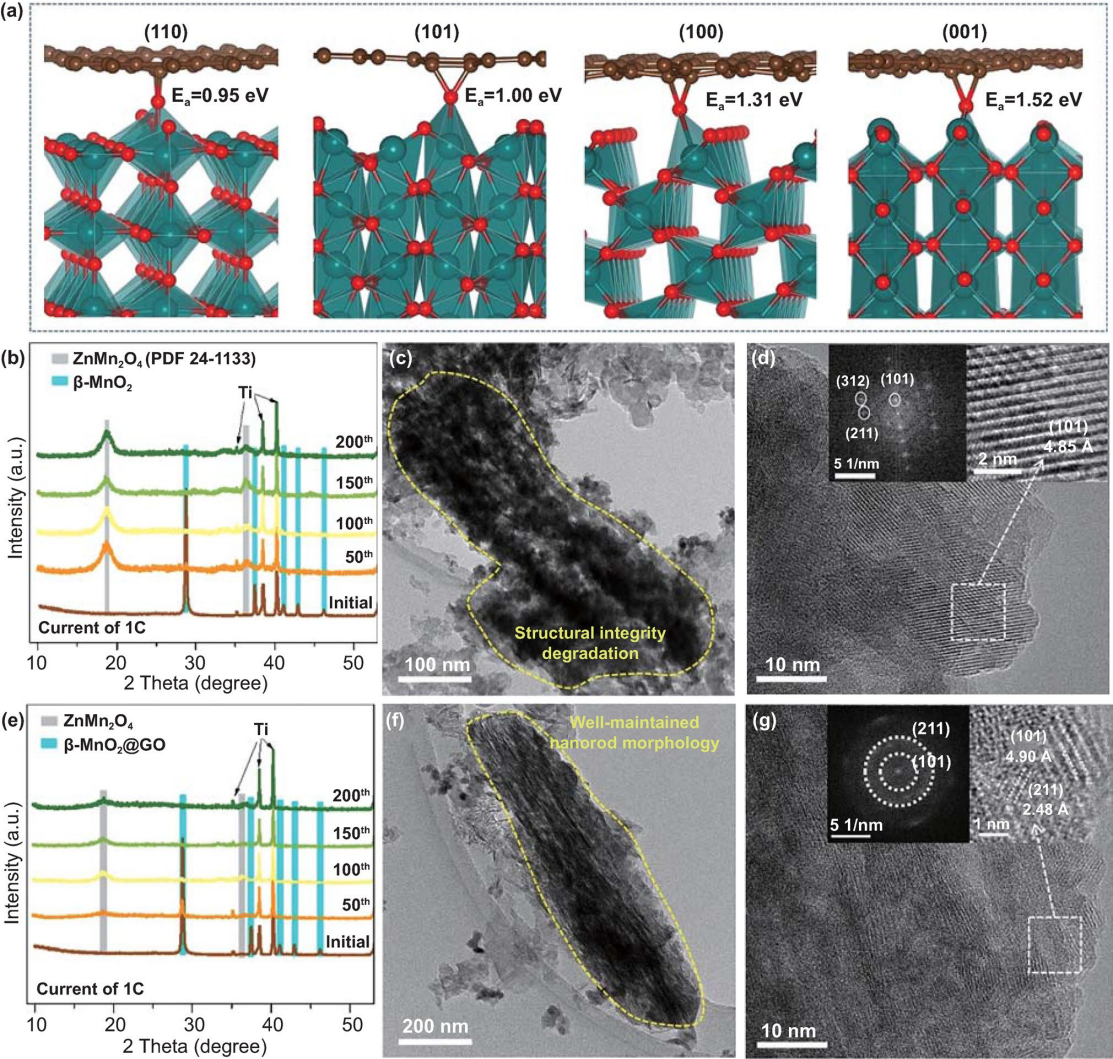
**a** DFT calculated binding configuration and adsorption energies (*E*_a_) of GO on β-MnO_2_ (110), (101), (100), and (001) terraces with a surface V_O_. **b** XRD patterns of β-MnO_2_ electrodes of pristine and at charged state after 50, 100, 150, and 200 cycles at current of 1C. **c**, **d** TEM morphologies of active material in β-MnO_2_ electrode after 200 cycles at current of 1C, showing a degradation on structural integrity, and the corresponding HRTEM images (inset, diffraction pattern of ZnMn_2_O_4_ spinel). **e** XRD patterns of β-MnO_2_@GO electrodes of pristine and at charged state after 50, 100, 150, and 200 cycles at current of 1C. **f**, **g** TEM morphologies of active material in β- MnO_2_@GO electrode after 200 cycles at current of 1C, showing a well-maintained nanorod morphology, and the corresponding HRTEM images (inset, diffraction rings showing (211) and (101) planes of nanocrystalline Zn_x_Mn_2_O_4_ spinel)

As illustrated in Fig. 2d, e, the GO wrapping can dramatically enhance the cycling stability. In this part, the mechanism of such enhancement is comprehensively investigated. DFT calculations are applied to reveal the interaction between β-MnO_2_ and GO. In the absence of an ether oxygen on GO, the graphene layer is weakly bound to the β-MnO_2_ surfaces via van der Waals forces, with adsorption energies ranging from 0.21 to 0.44 eV (Fig. S16). Surface V_O_ of β-MnO_2_ cannot contribute to sufficiently strong interaction. However, when there exist surface V_O_ and an ether oxygen in the vicinity, chemical bonding is established featuring Mn–O–C configuration, which pushes the adsorption energy to as high as 0.95–1.52 eV (Fig. 5a). It can be drawn that V_O_ in β-MnO_2_ and ether oxygen on GO work in synergy to achieve an intimate self-assembled wrapping of GO on β-MnO_2_, which provides a direct physical barrier rendering the Mn ions tightly confined beneath the surfaces even at low valence states.

Figure 5b shows the structure evolution of the β-MnO_2_ electrodes. During cycling, the characteristic peaks of β-MnO_2_ at 28.7° and 37.5° decrease gradually and disappear after 50 cycles. Meanwhile, the characteristic peaks of ZnMn_2_O_4_ at 18.7° and 36.3° emerge and increase gradually upon cycling. These results indicate a progressive structure evolution from bulk β-MnO_2_ to ZnMn_2_O_4_ spinel. Figure 5c, d shows the TEM/HRTEM images and correlated diffraction pattern of the active material in β-MnO_2_ after 200 cycles. We observe a severe degradation on the structural integrity of β-MnO_2_, and the active material has completely converted into a bulk (or long-range-ordered) Zn_*x*_Mn_2_O_4_ spinel (*x* = 1.000, from ICP result), as confirmed by the clear lattice fringe of (101) plane, as well as the apparent diffraction spots representing the (101), (211), and (312) plane (diffraction pattern shown in the inset of Fig. 5d). The TEM EDS mapping in Fig. S17 further indicates the uniform distribution of Zn, O, and Mn elements, substantiating the generation of ZnMn_2_O_4_ spinel after long-term cycling.

For β-MnO_2_@GO, the structural evolution is different from that of the β-MnO_2_, as illustrated in Fig. 5e. The characteristic peaks of β-MnO_2_@GO retain well after 100 cycles, demonstrating the beneficial effect of GO wrapping on stabilizing the pristine lattice framework. The relative intensities of the characteristic peaks of ZnMn_2_O_4_ in β-MnO_2_@GO electrode are much lower than that in β-MnO_2_ electrode, indicating that GO wrapping can effectively inhibit ZnMn_2_O_4_ accumulation upon long-term cycling. The nanorod morphology of β-MnO_2_@GO is well preserved even after 200 cycles (Fig. 5f), showing an enhanced structural integrity. Figure 5g shows the HRTEM morphologies and correlated diffraction patterns of the active material, which shows vague lattice fringes referring to the (101) and (211) planes of Zn_*x*_Mn_2_O_4_ (*x* = 0.846, from ICP result) spinel with lattice spacing of ~ 4.90 Å and ~ 2.48 Å, respectively. The correlated diffraction pattern shows two diffraction rings (inset in Fig. 5g), indicating the nanocrystalline (or short-term ordered) feature that favors proton intercalation/conversion reactions. EDS mapping results show a uniformly distributed Zn, O, and Mn elements in the active material of β-MnO_2_@GO electrode after 200 cycles (Fig. S18), confirming the generation of nanocrystalline ZnMn_2_O_4_.

Overall, the combinatorial incorporation of V_O_ and SC in β-MnO_2_ could help in achieving better electrochemical performance on the following mechanistic aspects: (1) both V_O_ and GO wrapping could facilitate electron transport; (2) intimate adhesion of GO on the defective surface could pose barrier to the dissolution of Mn ions; (3) combination of V_O_ and GO wrapping can retard the Zn_*x*_Mn_2_O_4_ accumulation and regulate the structural evolution.

## Conclusions

In this work, the concurrent application of both defect engineering and interfacial optimization to a manganese oxide electrode for AZIBs is for the first time demonstrated. Oxygen vacancies are spontaneously introduced into β-MnO_2_ during its synthesis in the presence of GO that eventually builds a coating layer on the active material. For the as-prepared oxygen-deficient β-MnO_2_@GO cathode, the successful suppression of Mn dissolution during electrochemical cycling is made possible, along with an apparent enhancement in charge/discharge kinetics. This electrode delivers a capacity of ~ 129.6 mAh g^−1^ even after 2000 cycles at a current rate of 4C, which is much superior than that of pristine β-MnO_2_ electrode. The excellent cycle stability is rooted in the strong binding between the surface V_O_ and ether oxygen on GO, as well as the regulated structural evolution into the nanocrystalline Zn_*x*_Mn_2_O_4_ phase. The results in this work highlight the advantages of integrating multiple strategies in the design of AZIB electrodes via bottom-up synthetic approaches, which will cast light on the feasibility of AZIBs in meeting the high-rate and long-life requirements for large-scale energy storage applications.

## Supplementary Information

Below is the link to the electronic supplementary material.

Supplementary file1 (PDF 1519 KB)
