# Supplementary material for: Oxygen-Deficient β-MnO2@Graphene Oxide Cathode for High-Rate and Long-Life Aqueous Zinc Ion Batteries
Source: Nanomicro Lett. 2021 Aug 13;13:173. doi: 10.1007/s40820-021-00691-7 (PMC8363675; doi:10.1007/s40820-021-00691-7)
Supplement: Supplementary file 1 — Supplementary file1 (PDF 1519 KB) [file 40820_2021_691_MOESM1_ESM.pdf]

Supporting Information for

Oxygen-Deficient β-MnO_2@Graphene Oxide Cathode for High-Rate and Long-Life Aqueous Zinc Ion Batteries

Shouxiang Ding[1], Mingzheng Zhang[1], Runzhi Qin[1], Jianjun Fang[1], Hengyu Ren[1], Haocong Yi[1], Lele Liu[1], Wenguang Zhao[1], Yang Li[1], Lu Yao[1], Shunning Li[1,] *, Qinghe Zhao[1,] *, Feng Pan[1,] *

[1]School of Advanced Materials, Peking University Shenzhen Graduate School, Shenzhen 518055, P. R. China

*Corresponding authors. E-mail: lisn@pku.edu.cn (Shunning Li); zhaoqh@pku.edu.cn (Qinghe Zhao); panfeng@pkusz.edu.cn (Feng Pan)

S1 DFT Calculation Methods

Valence electron configurations for elemental constituents were as follows: C-$2s^22p^2$, O-$2s^22p^4$ and Mn-$3d^64s^1$. The cutoff energy of 500 eV was chosen to optimize the geometry of all structural configurations and calculate the corresponding electronic structures. In the process of electronic and geometric optimizations, energies and residual forces were converged to 10^{-5} eV and 0.02 eV $Å^{-1}$, respectively. In the PBEsol+U(+J) calculations, the values of U=2.8eV and J=1.2eV were adopted from the previous work [S1]. The PBEsol + U(+J) scheme predicted a gap of 0.25 eV, which agrees well with the experimental result of 0.27 eV [S2]. Following the work of Kresse et al. [S3], we applied a collinear antiferromagnetic ordering for Mn ions in all our calculations.

To investigate the electronic structures in the presence of V_O, we constructed a 2 × 2 × 2 supercell of β-MnO_2. A 8 × 8 × 11 mesh of *k*-point grid determined by Monkhorst-Pack [S4] method was employed. According to the Wulff's construction of the equilibrium morphology calculated by Thomas and Khomotso [S5], the (110), (100), (101) and (001) surfaces of β-MnO_2 were selected to determine the difference in adsorption configurations and energies between graphene and graphene oxide. The lattice parameters of the β-MnO_2 slab were fixed to bulk values, while the structure of the adsorbed graphene was slightly stretched or compressed to attain a commensurate supercell. Tables S4 and S5 summarize the lattice mismatch for the corresponding configurations.

S2 Diffusion Kinetics Calculation based on GITT Curves

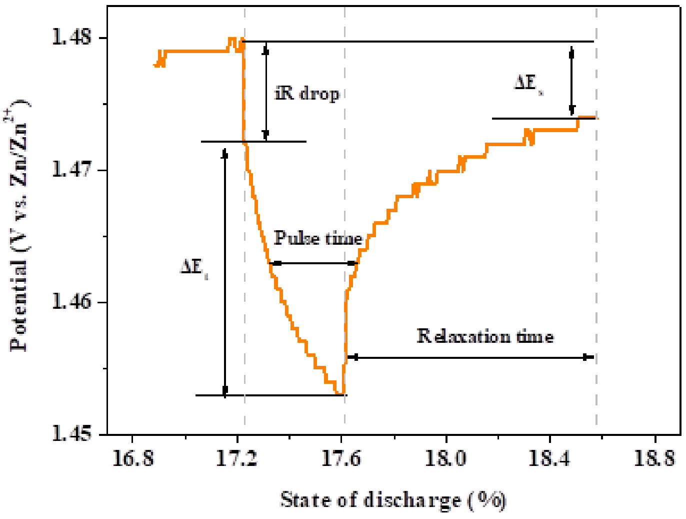

Scheme S1 Schematic illustration for calculation of diffusion kinetics based on GITT curves

The solid diffusion coefficient of layered MnO_2 cathodes is conducted by Galvanostatic Intermittent Titration Technique (GITT) and calculated based on Eq. (S1),

$$D = \frac{4L^2}{\pi\tau}\left(\frac{\Delta E_s}{\Delta E_t}\right)^2 \quad \text{(S1)}$$

where ΔE_s (V) is the steady state potential change by the current pulse, and ΔE_t (V) expresses the potential change during the constant current pulse after eliminating the iR drop. L is ion diffusion length (cm), for compact electrode, it is equal to the electrode thickness. τ is Relaxation time.

S3 Supplementary Figures and Tables

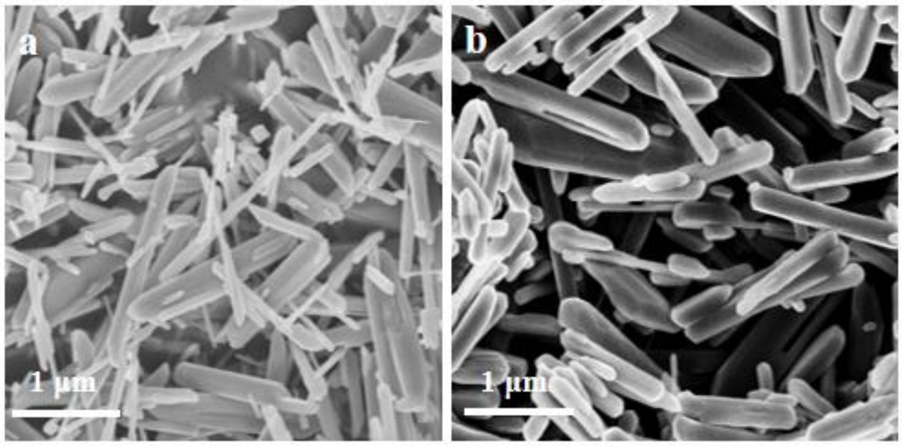

Fig. S1 SEM morphologies of β-MnO_2 and β-MnO_2@GO

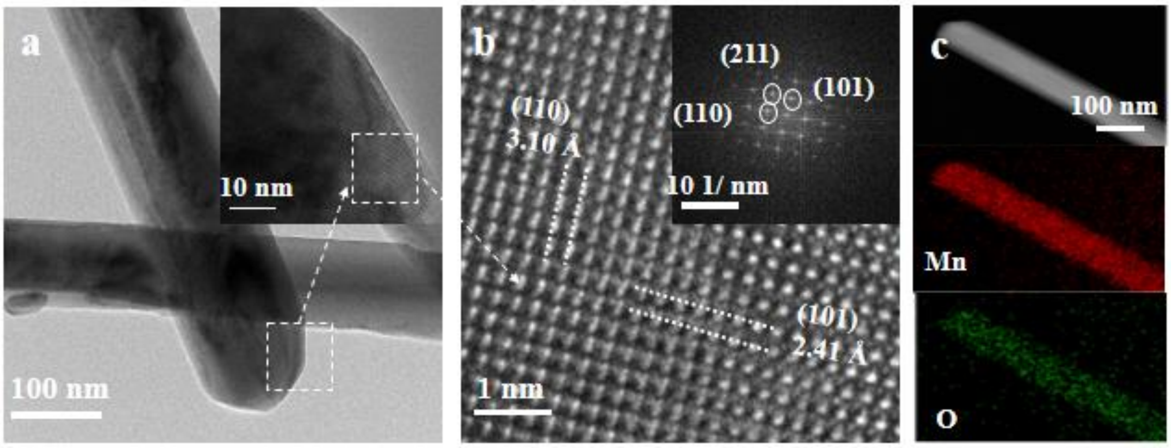

Fig. S2 TEM/ HRTEM images and EDS-mapping results of β-MnO_2

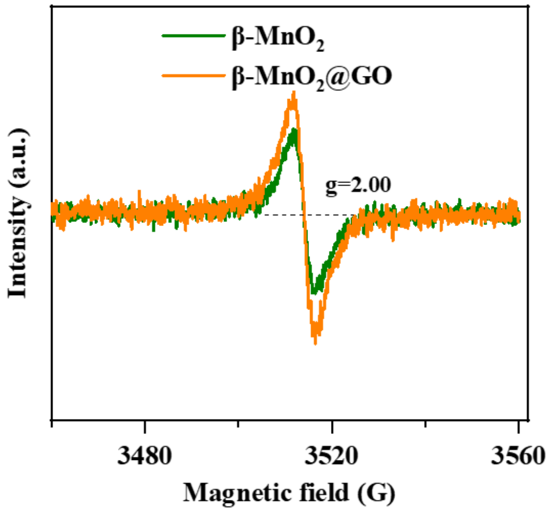

Fig. S3 EPR spectra of β-MnO_2 and β-MnO_2@GO

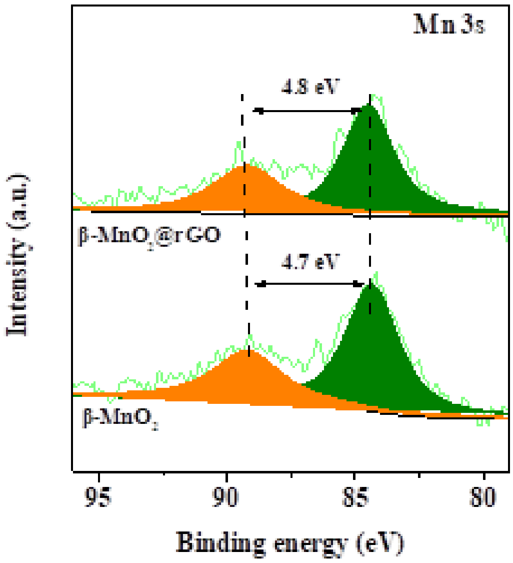

Fig. S4 XPS spectra of Mn 3s spectra of β-MnO_2@GO and β-MnO_2

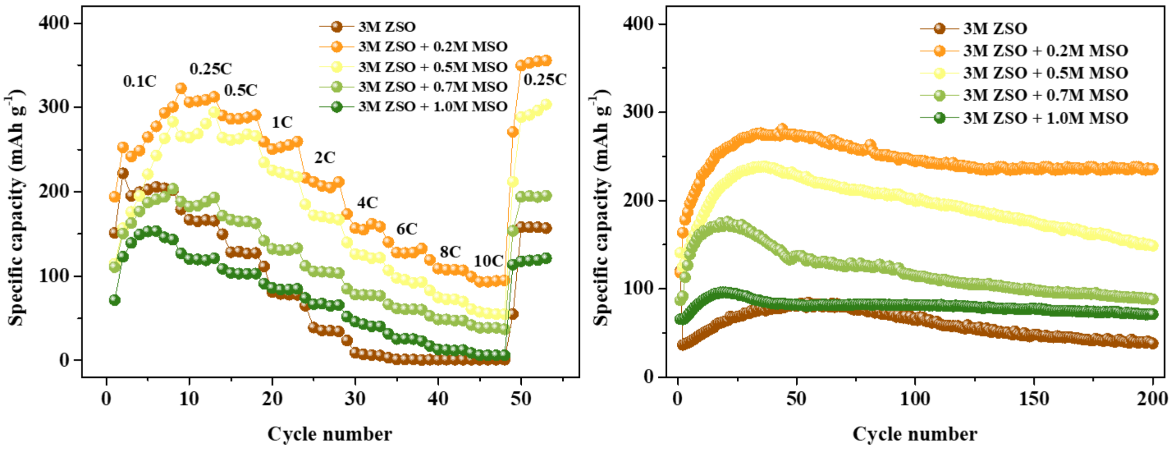

Fig. S5 Comparison of rate and cycling performances of β-MnO_2@GO electrodes in electrolytes with different Mn^{2+} concentrations. ZSO represents the $ZnSO_4$, and MSO represents the $MnSO_4$

Table S1 Rate performances of manganese oxides in previous reports

Materials	Rate performance	References
β-MnO_2@GO	322.6 mAh g^{-1} at 0.1C, 94.6 mAh g^{-1} at 10C, and with no capacity fading in 2000 cycles at 4C.	*This work*
yolk-shell MnO_2@C	239 mAh g^{-1} at 0.1 A g^{-1}, 91 mAh g^{-1} at 2.0 A g^{-1}, 93% capacity retention in 1000 cycles at 1 A g^{-1}	*ChemElectroChem*, 2020, 7: 1166–1171
MnO_2/acid-treated CNT Nanocomposites	197 mAh g^{-1} at 0.1 A g^{-1}, 87 mAh g^{-1} at 5.0 A g^{-1}, with 90% capacity retention in 500 cycles at 5 A g^{-1}	*Electrochimica Acta*, 2014, 133: 254–261
β-MnO_2@C hybrids	130 mAh g^{-1} at 0.1 A g^{-1}, 40 mAh g^{-1} at 2.0 A g^{-1}, and with no capacity fading in 250 cycles at 0.2 A g^{-1}	*Journal of Alloys and Compounds*, 2020, 827: 154273
MnO_x/PPy	408 mAh g^{-1} at 1C, 67 mAh g^{-1} at 10C, and with 70% capacity retention in 2500 cycles at 5C	*ACS Appl. Mater. Interfaces,* 2020, 12, 8: 9347–9354
polyfurfural/MnO_2	500 mAh g^{-1} at 0.05 A g^{-1}, 95 mAh g^{-1} at 0.5 A g^{-1}, and with 90% capacity retention in 160 cycles at 0.2 A g^{-1}	*ACS Appl. Mater. Interfaces,* 2020, 12, 32: 36072–36081
β-MnO_2	300 mAh g^{-1} at 0.3 A g^{-1}, 170 mAh g^{-1} at 0.9 A g^{-1}, and with 35% capacity retention in 200 cycles at 0.2 A g^{-1}	*ACS Appl. Mater. Interfaces*, 2020, 12: 12834−12846
α-MnO_2	215 mAh g^{-1} at 0.5C, 126 mAh g^{-1} at 10C, and and with 68% capacity retention in 100 cycles at 6C	*Angew. Chem. Int. Ed.* 2012, 51: 933 –935
α-MnO_2 nanorod	353 mAh g^{-1} at 0.016 A g^{-1}, 43 mAh g^{-1} at 1.333 A g^{-1}, and with 60% capacity retention in 1000 cycles at 83 mA g^{-1}	*Journal of Power Sources,* 2015, 288: 320-327

β-MnO_2	205 mAh g^{-1} at 0.25 A g^{-1}, 21 mAh g^{-1} at 2.0 A g^{-1}, and with 50% capacity retention in 1000 cycles at 0.2 A g^{-1}	*Journal of Energy Chemistry* 2021, 56: 365-373
α-MnO_2	260 mAh g^{-1} at 0.1 A g^{-1}, 130 mAh g^{-1} at 2.0 A g^{-1}, and with 50% capacity retention in 300 cycles at 0.5 A g^{-1}	*Small,* 2020, 1905842
$K_xMn_{8-x}O_{16}$	130 mAh g^{-1} at 0.05 A g^{-1}, 78 mAh g^{-1} at 0.2 A g^{-1}, and with 90% capacity retention in 300 cycles at 0.1 A g^{-1}	*Journal of Energy Storage,* 2020, 27, 101139
Ce-doped MnO_2	250 mAh g^{-1} at 0.5C, 80 mAh g^{-1} at 5C, and and with 60% capacity retention in 100 cycles at 5C	*J. Phys. Chem. C,* 2019, 123, 22735−22741

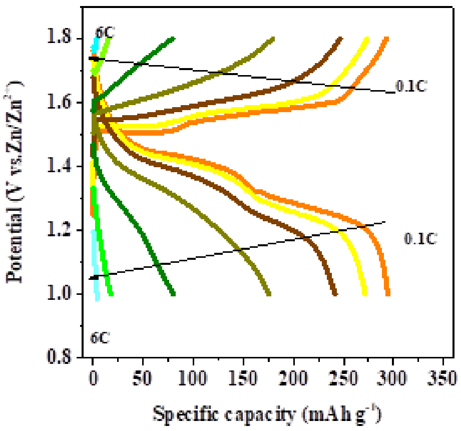

Fig. S6 GCD curves of the β-MnO_2 electrode at different rate currents

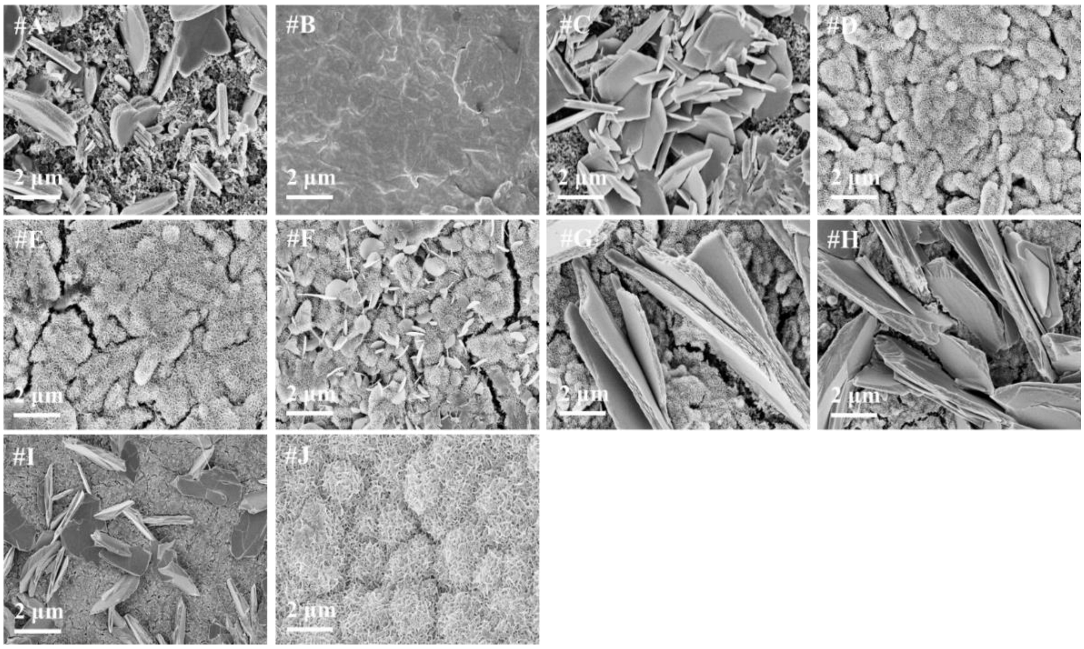

Fig. S7 Electrode surface morphology of the β-MnO_2@GO electrodes at different charge/discharge states

As shown in **Fig. S7**, upon discharging, a large number of flake-like products appear, which belong to the zinc sulfate hydroxide hydrate (ZSH, $Zn_4(OH)_6 \cdot ZnSO_4 \cdot xH_2O$, JCPDS #44-0673 and 39-0688). This can be ascribed to the H^+ insertion into MnO_2 forming MnOOH and β-H_xMnO_2, thus leaving OH^- in the electrolyte that interact with $ZnSO_4$ and H_2O to conform the ZSH attached on the surface of electrodes. The difference in ZSH by-products may be attributed to the loss of structural water during drying procedure. It is clearly observed from the SEM images that the ZSH phase appears on the surface of electrodes at discharge and displays a flake-like morphology. The reversible formation and elimination of ZSH during electrochemical reaction suggests that H^+ can insert and extract reversibly.

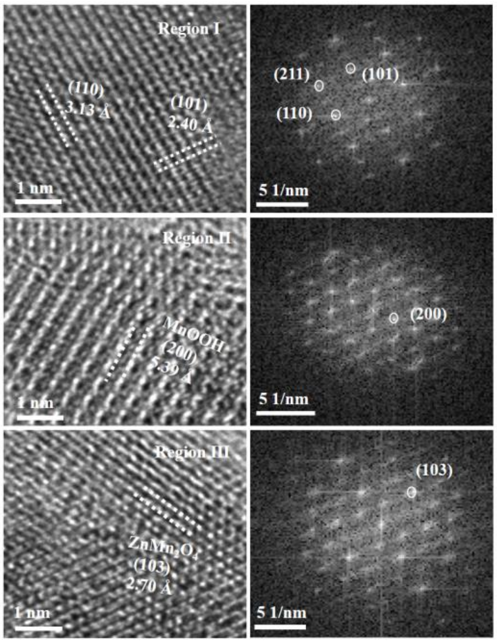

Fig. S8 Detailed analyses of the diffraction patterns in regions I to III of β-MnO_2@GO at discharged state in Figure 3e to 3g

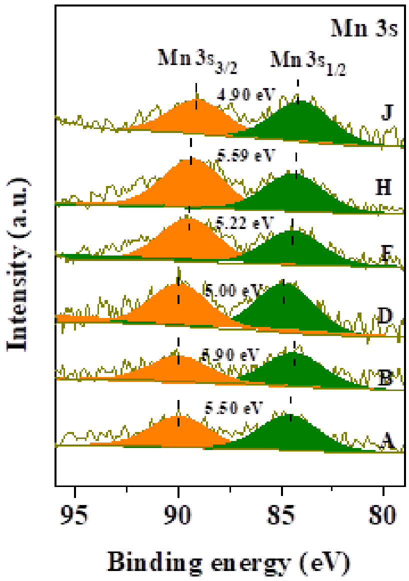

Fig. S9 XPS analyses of Mn 3s spectra of β-MnO_2@GO at different charge/discharge states

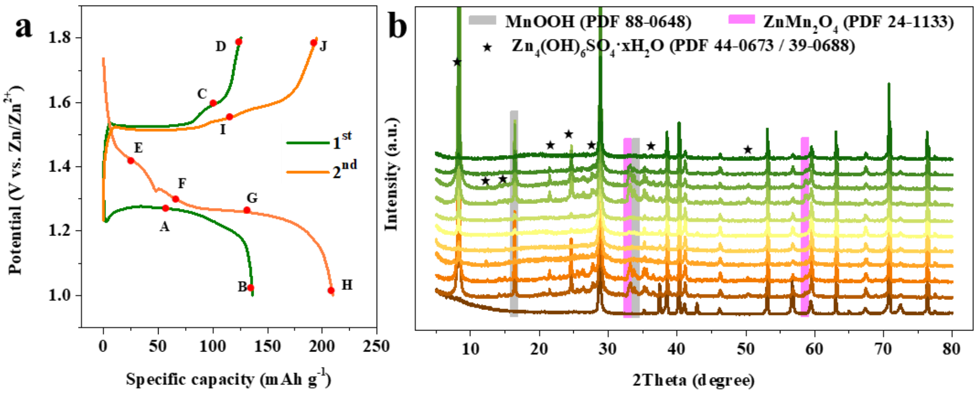

Fig. S10 Structural evolution of β-MnO2 during the process of cycling. (**a**) Electrochemical profile of β-MnO2 cathode at 0.1 C. (**b**) Ex suit XRD patterns at selected points at different states

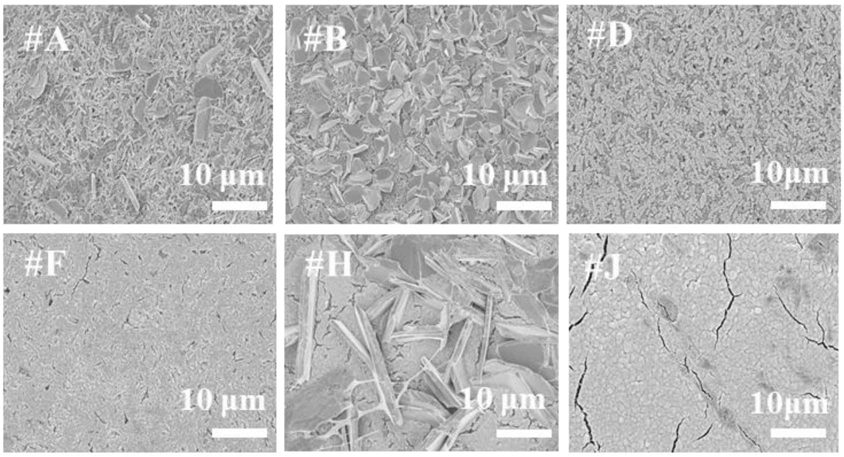

Fig. S11 SEM morphologies of β-MnO2 cathodes during the process of cycling at varied states

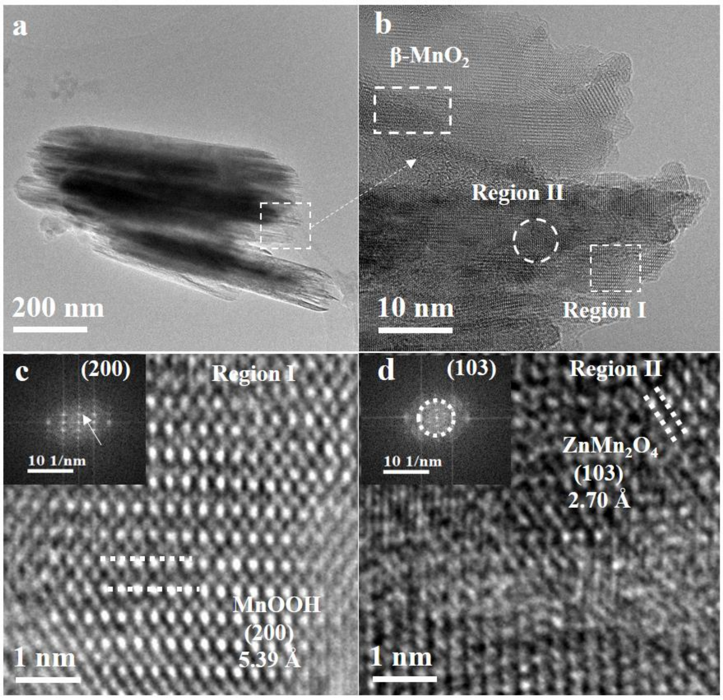

Fig. S12 TEM/HRTEM analyses of β-MnO2 at the discharged state

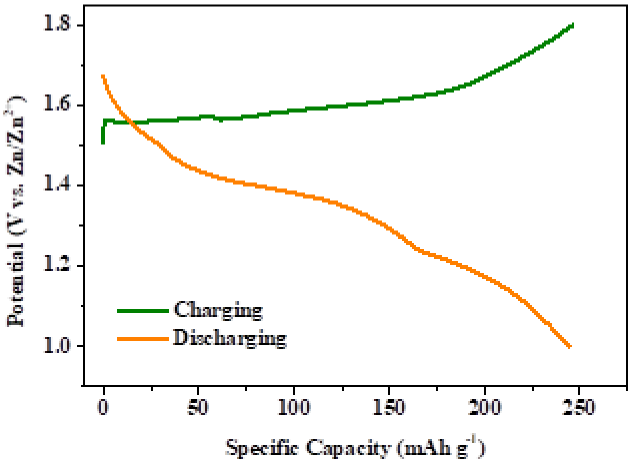

Fig. S5 GCD curves of β-MnO_2@GO after 100 cycles at current of 1C

Table S2 ICP results of β-MnO_2@GO at the 100th cycle

Sample	CC/Zn	CC/Mn	Zn/Mn
100-cha	1.75	8.22	0.179
100-dis	0.87	3.87	0.193

Table S3 Contributions of proton and Zn^{2+} ions on electrode capacity delivery after long-term cycle

Sample	Capacity	ΔZn/Mn	e^-	ΔH/Mn
β-MnO_2@GO	244.42 mAh g^{-1}	0.014	0.793	0.765

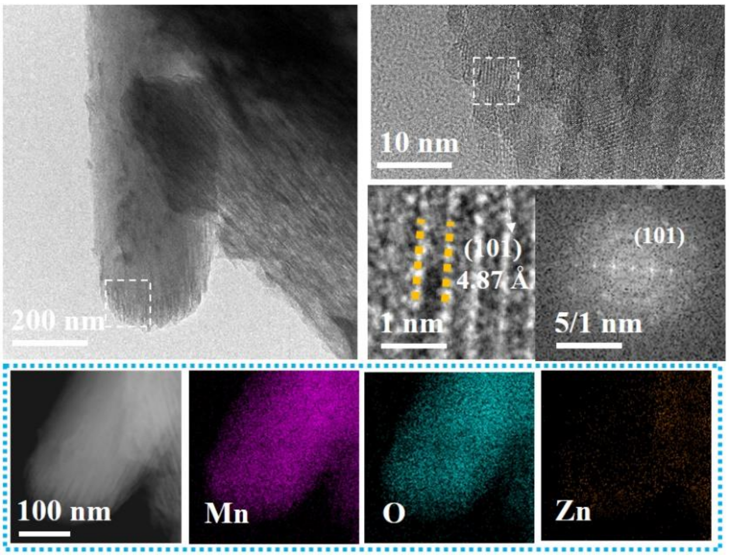

Fig. S14 HRTEM morphologies, diffraction patterns and EDS-mapping distributions of β-MnO_2@GO after 100 cycles (at the charged state), which indicates a formation of nanocrystalline $Zn_xMn_2O_4$ spinel phase

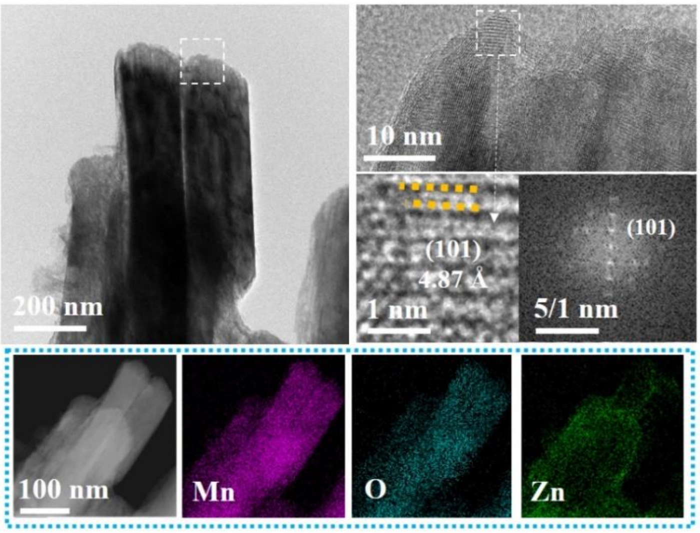

Fig. S15 HRTEM morphologies, diffraction patterns and EDS-mapping distributions of β-MnO_2@GO after 100 cycles (at the discharged state), which indicates a formation of nanocrystalline $Zn_xMn_2O_4$ spinel phase

Table S4 Lattice mismatch between β-MnO_2 and GO in direction A of the supercell

hkl	A_{hkl}	n_{hkl}	n_{gr}	Mismatch = $(n_{gr}\times A_{gr}-n_{hkl}\times A_{hkl})/(n_g\times A_{gr})$, %
110	2.867	3	2	-0.85
100	4.368	1	1	-2.42
101	4.368	1	1	-2.42
001	4.368	1	1	-2.42

Table S5 Lattice mismatch between β-MnO_2 and GO in direction B of the supercell

hkl	B_{hkl}	n_{hkl}	n_{gr}	Mismatch = $(N_{gr}\times B_{gr}-N_{hkl}\times B_{hkl})/(N_g\times B_{gr})$, %
110	6.177	2	5	-0.63
100	2.867	6	7	0.09
101	5.225	1	2	-6.40
001	4.368	4	7	-1.65

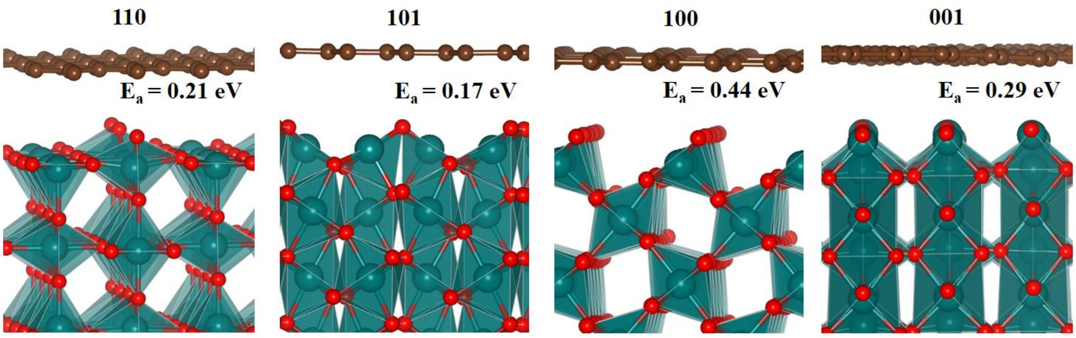

Fig. S16 DFT evaluation of the van der Waals interaction between graphene and β-MnO_2 (110), (101), (100) and (001) terraces with surface V_O. *E*a represents the adsorption energy

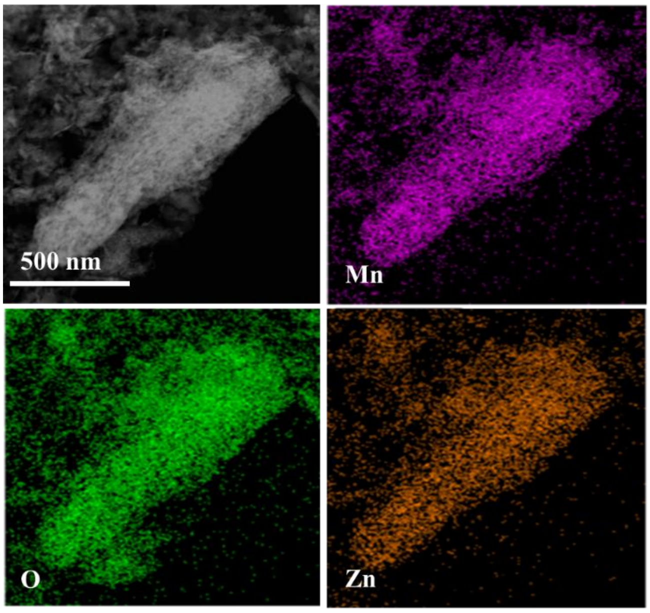

Fig. S17 EDS-mapping results showing the uniform distribution of Zn, O, and Mn elements in active material of β-MnO_2 electrode at 200 cycles at 1C

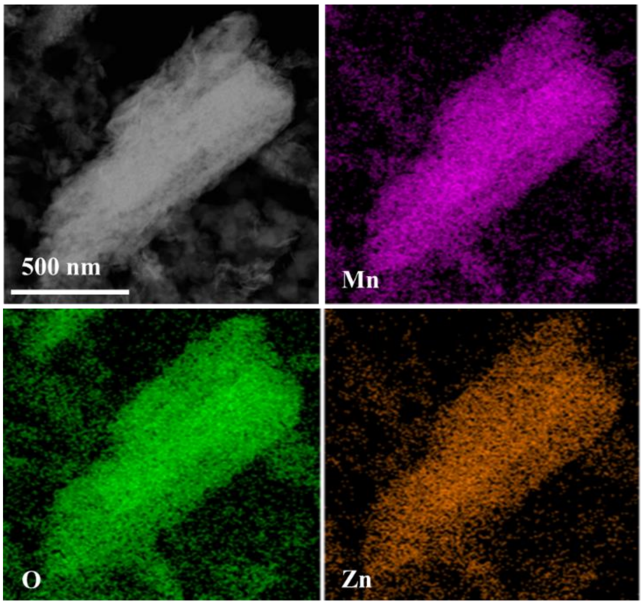

Fig. S18 EDS-mapping results showing the uniform distribution of Zn, O, and Mn elements in active material of β-MnO_2@GO electrode at 200 cycles at 1C

Supplementary References

[S1] E. Cockayne, L. Li, First-principles DFT+U studies of the atomic, electronic, and magnetic structure of α-MnO_2 (cryptomelane). Chem. Phys. Lett. **554**, 53-58 (2012). https://doi.org/10.1016/j.cplett.2012.06.061

[S2] A. Islam, R. Islam, K. Khan, Studies on the thermoelectric effect in semiconducting MnO_2 thin films. J. Mater. Sci. Mater. Electron. **16**(4), 203-207 (2005). https://doi.org/10.1007/s10854-

005-0766-1

[S3] C. Franchini, R. Podloucky, J. Paier, M. Marsman, G. Kresse, Ground-state properties of multivalent manganese oxides: Density functional and hybrid density functional calculations. Phys. Rev. B **75**(19), 195128(2007). https://doi.org/10.1103/PhysRevB.75.195128

[S4] D. Chadi, Special points for Brillouin-zone integrations. Phys. Rev. B **16**(4), 1746-1747 (1977). https://doi.org/10.1103/PhysRevB.16.1746

[S5] T. Mellan, K. Maenetja, P. Ngoepe, S. Woodley, C. Catlow, R. Grau-Crespo, Lithium and oxygen adsorption at the β-MnO_2 (110) surface. J. Mater. Chem. A **1**(47), 14879-14887 (2013). https://doi.org/ 10.1039/c3ta13559d
